# Early Post‐Kidney Transplant Treatment With Roxadustat Ameliorates Anemia and Reduces Blood Transfusion Needs in Inflamed Patients: A Case‐Matched Study

**DOI:** 10.1111/ctr.70617

**Published:** 2026-07-08

**Authors:** Paolo Randone, Laura Valenza, Alberto Mella, Fabrizio Fop, Caterina Dolla, Ester Gallo, Ana Maria Manzione, Silvia Mingozzi, Rita Tarragoni, Enrico Sanna, Luigi Biancone

**Affiliations:** ^1^ Renal Transplant Center “A. Vercellone”, Nephrology, Dialysis and Renal Transplant Division, Department of Medical Sciences “Città Della Salute e della Scienza” Hospital, University of Turin Turin Italy

**Keywords:** blood transfusion, erythropoiesis‐stimulating agents, post‐transplant anemia, roxadustat

## Abstract

**Background:**

Roxadustat is an oral hypoxia‐inducible factor prolyl‐hydroxylase inhibitor. Recent studies have shown no inferiority of roxadustat compared to erythropoietin in chronic kidney disease and in post‐transplant patients. We evaluated roxadustat versus erythropoiesis‐stimulating agents (ESAs) in very early posttransplant patients.

**Methods:**

We performed a retrospective analysis of the clinical course and laboratory changes in posttransplant patients treated with roxadustat (16 patients) or a high‐dose erythropoiesis‐stimulating agent (ESA) (16 patients) for anemia between March and September 2025. We compared haemoglobin, creatinine, lipid levels, and iron status. The therapy was started after the kidney transplant, respectively, after a median of 3.5 and two days. Every patient was followed up at two weeks, one month, two months, and three months.

**Results:**

Patients treated with roxadustat showed a significantly greater and faster increase in Hb levels at 2 weeks (*p* = 0.047) than the ESA group. After 2 weeks, we observed a significant difference in the need for blood transfusions between the groups (*p* = 0.029; 18.8% in the roxadustat group vs. 62.5% in the ESA group). No significant differences were observed in iron or inflammation status during the study. The roxadustat group achieved a significantly better lipid profile after one month (*p* < 0.05 for cholesterol and LDL). During the follow‐up, no adverse events related to roxadustat were reported.

**Conclusion:**

Administration of oral roxadustat in very early post‐transplant patients is effective and safe, rapidly improving anemia and reducing the need for transfusions.

**Abbreviations**
AMR: Antibody‐Mediated Rejection
ATG: Anti‐Thymocyte Globulin
BL‐R: Borderline Rejection
CaTCMR: Chronic‐Active T‐cell mediated rejection
CKD: Chronic Kidney Disease
CKD‐EPI: Chronic Kidney Disease Epidemiology Collaboration
CNI: Calcineurin Inhibitor
CRP: C‐Reactive Protein
DBD: Donation after Brain Death
DCD: Donation after Circulatory Death
DGF: Delayed Graft Function
DSA: Donor Specific Antibodies
EPO: Erythropoetin
ESA: Erythropoiesis‐stimulating agent
HIF: Hypoxia‐Inducible Factor
HIF‐PH: Hypoxia‐Inducible Factor Prolyl‐Hydroxylase Inhibitor
IVIG: Intravenous Immunoglobulin
KTRs: Kidney Transplant Recipients
MP: Methylprednisolone
PTA: Post‐Transplant Anemia
sCr: Serum Creatinine
TCMR: T‐cell‐mediated rejection
TIA: Transient Ischemic Attack
TIBC: Total Iron Binding Capacity
TSAT: Transferrin Saturation
UTI: Urinary Tract Infection

## 1 Introduction

After a kidney transplantation, several causes can aggravate anemia due to chronic kidney disease (CKD). Surgical blood loss, immunosuppressive drugs, infections (CMV, parvovirus B19), and high inflammatory status are the most frequent reasons to cause anemia in almost 90% of kidney transplanted patients (KTRs) in the first month after transplantation [1, 2, 3, 4]. Posttransplant anemia (PTA) was defined in 2000 as hemoglobin (Hb) concentration <130 g/L in men and Hb <120 g/L in women [5].

Conventional treatment for anemia is based on erythropoiesis‐stimulating agents (ESAs) and supplementation with hematopoietic nutrients (e.g., iron, folic acid, or vitamin B12). Unfortunately, despite the use of large doses, the response is ineffective in patients with a high inflammatory state [6].

Recently, new studies have demonstrated that a novel agent, hypoxia‐inducible factor prolyl hydroxylase inhibitor (HIF‐PHI), is safe and effective for renal anemia. The HIF pathway increases endogenous erythropoietin production. These new drugs inhibit HIF‐PHI, the enzyme that degrades the transcription factor HIF, thereby increasing its half‐life. It has also been demonstrated that HIF‐PHI agents are effective even in cases with high inflammatory status [7, 8].

Current literature demonstrates no inferiority between HIF‐PHI agents and ESA. In particular, roxadustat, the first oral HIF‐HI, demonstrated safety and efficacy, and was noninferior to ESA for treating anemia in CKD [7, 8] and PTA [9, 10, 11, 12]. There is no consensus on the start timing of roxadustat in PTA. Most studies began more than six months after transplantation, whereas only two studies by Li et al. [9]. and Guenal et al. [13]. began earlier (median 15 days after transplantation). In accordance with these works, to achieve maximal benefit from roxadustat, initiation should be considered when the patient's inflammatory state is highest to minimize the risk of transfusion. In this study, we compared the efficacy and safety of high‐dose ESA and standard‐dose roxadustat in KTRs who initiated therapy a few days after transplantation. We analyzed not only the variation in Hb status but also the differences in blood transfusion requirements between the two groups.

## 2 Materials and Methods

### 2.1 Studied Population

Following the approval of Roxadustat by the Italian Regulatory Agency (AIFA), regardless of prior erythropoietin (EPO) use, we began prescribing roxadustat to early post‐transplant patients at clinicians' discretion. Clinical and renal functional data were extracted from the medical charts of all KTs performed at the Turin University Renal Transplant Center “A. Vercellone” March 2025–September 2025. Each recipient was followed by the transplant center with at least one annual visit and by the local nephrologist (11 peripheral centers covering most of the Piedmont region) for periodic follow‐up.

Patients treated with Roxadustat were compared with a population of consecutive KTRs who received high‐dose ESA immediately after transplantation and had normal ferritin levels, as achieved during dialysis, along with continuous iron supplementation and a stable inflammatory status as observed in the immediate post‐transplant period (ferritin levels > 300 µg/L). Both patient groups presented with a PTA and an Hb < 12 g/dL.

To avoid selection bias, we excluded from both groups patients with bleeding complications during or immediately after the transplant. Hb values were analyzed before and after the transplant, along with serum creatinine (sCr), estimated glomerular filtration rate (eGFR), lipids, and iron status. Data were reported before transplantation, at the time of transplantation, and at two weeks, one month, two months, and three months posttransplantation. All patients started the therapy early after the transplant (<7 days). Patients were eligible for blood transfusion if their Hb was <7.5 g/dL (no history of cardiovascular disease) or <9 g/dL (history of cardiovascular disease), or if they developed symptoms. Patients in both groups received endovenous iron if ferritin <200 µg/L and TSAT <20% (ferric carboxymaltose 500 mg, one injection; three patients in both groups). We repeated the administration once a week during the hospitalization, and at discharge, the endovenous iron administration was discontinued. Vitamin B12 and folate were supplemented according to deficiency, while statins and omega‐3 were administered based on lipid status at discharge. In both groups, therapy was reduced or discontinued if the Hb level exceeded 13 g/dL. Hb trends were analyzed between the two groups at the most recent visit for each patient in the study. Additionally, potential risk factors were analyzed, including thrombotic risk (thrombotic events), cardiovascular risk (history of stroke, arrhythmias), prior infection events (pyelonephritis, pneumonitis, UTIs), oncological risk (previous neoplasia), and immunological risk (prior glomerulonephritis, other transplants). Clinical and laboratory data were extracted from electronic medical records. This retrospective study was approved by the Institutional Ethics Committee of the Città Della Salute e Della Scienza, Turin, Italy. All patients have signed a consent form to participate in the study (Protocol study number 00157/2019).

### 2.2 Statistical Analysis

Continuous variables were described using median and interquartile range (IQR) according to their non‐normal distribution. To compare independent groups, we used the Mann‐Whitney test. The difference between before‐and‐after observations was analyzed using a paired Student's t‐test or Wilcoxon test. Categorical variables were presented as fractions, and Pearson's χ2 test or, for small samples, Fisher's exact test was employed to compare groups. Odds ratios (ORs) with 95% Confidence Intervals were used as measures of relative risk. Scatter plots and box plots were used to explore relationships among variables visually. The significance level for all tests was set at *α* < 0.05. All statistical analyses were performed using SPSS (IBM Corp. Released 2022. IBM SPSS Statistics for Windows, Version 29.0.1. Armonk, NY: IBM Corp).

## 3 Results

### 3.1 Population Characteristics and Therapy

We administered roxadustat to 16 consecutive KTRs (dose: 70–100 mg, three times per week, according to body weight), and we compared them with 16 consecutive KTRs treated with high‐dose EPO (Erythropoietin zeta) at 150 Units/kg subcutaneously three times per week (10,000 Units subcutaneously three times per week). Comparisons of baseline characteristics and laboratory data, both before and at the start of therapy, between the two groups are presented in Table 1. No significant demographic differences are present between the two groups. There is no difference between the two groups in baseline oncological, cardiovascular, infectious, and thromboembolic outcomes. All the patients received immunosuppressive therapy induction: Anti‐Thymocyte Globulin (ATG) 3‐4.5 mg/Kg, Methylprednisolone pulses, and immunosuppressive therapy maintenance with Tacrolimus (target level 8‐10 ng/mL), Mycophenolate Mofetil 1 g/day or Mycophenolic Acid 720 mg/day, and prednisone gradually tapered from 20 mg/day to 5 mg/day; no difference were found between the two groups. Before the kidney transplant and before starting roxadustat and ESA administration, Hb, MCV, MCH, MCHC, and RDW‐CV levels were comparable between the two groups (Table 1). Before beginning therapy, patients treated with roxadustat received more blood transfusions than those in the ESA group [6 (37.5% of roxadustat patients) vs. 1 (6.3% of ESA patients)]; this could depend on a significant difference in timing (p <0.001) of starting therapy (median 3.5 days for the roxadustat group, two days for the ESA group).

**TABLE 1:** Baseline and starting‐therapy data for the roxadustat and ESA groups. There were no differences between the two groups, except for the timing of therapy initiation (median 3.5 days in the roxadustat group and 2 days in the ESA group; *p* < 0.001).

Baseline	Roxadustat	ESA	*p* value
Patients n° (%)	16(50.0)	16(50.0)	
Gender f/m n° (%)	10(62.5)/ 6(37.5)	7(43.7)/9(56.2)	0.993
Race (%) White Asian African	13(81.3) 2(12.5) 1(6.3)	14(87.5) 0(0) 2(12.5)	
Age, median (25°–75°)	58.8(55.1–67.1)	55.8(40.3–62.1)	0.105
VPRA, median (25°–75°)	0(0)	20.8(0–45.3)	0.376
KDPI, median (25°–75°)	73(38.5–84.25)	56.5(35.25–80.75)	0.407
Time in dialysis in years, median (25°–75°)	2(1–4.75)	3(0.62–4)	0.849
Oncological Risk, n°	8	5	0.285
Cardiovascular Risk, n°	4	2	0.394
Trombo‐embolic Risk, n°	2	3	1.000
Infectious Risk, n°	2	4	0.654
Immunomediated Nephropathy, n°	2	6	0.220
Deceased Donor, n° (% by group)	16(100)	16(100)	1.000
Deceased Donor, n° (% by group)	16(100)	16(100)	1.000
Pre‐emptive recipients, n° (% by the group)	2(12.5)	3(18.8)	0.766
DCD donor, n° (% by the group)	4(25)	5(31.3)	0.694
DBD donor, n° (% by the group)	12(75)	11(68.8)	0.694
Delayed Graft Function (DGF), n° (% by the group)	3(18.8)	3(18.8)	1.000
BEFORE START 0 blood transfusions, n° (% by group) 1 blood transfusions, n° (% by group) 2 blood transfusions, n°(% by group) 3 blood transfusions, n°(% by group) 4 blood transfusions, n° (% by group)	10(62.5) 5(31.3) 0(0) 0(0) 1(6.3)	14(87.5) 0(0) 2(12.5) 0(0) 0(0)	
Pre‐Transplant			
Hb (g/dL), median (25°–75°)	11.5(10.7–13.2)	11.4(10.3–12.5)	0.386
HCT (%), median (25°–75°)	35.1(34.1–41.6)	35.5(31.3.5–40.9)	0.439
MCV (fL), median (25°–75°)	96(93–102)	99.5(92.2–100.7)	0.921
MCH (pg), median (25°–75°)	31.6(29.2–32.6)	31.2(30.1–32.7)	0.621
MCHC (g/L), median (25°–75°)	32.2(31.4–33.2)	31.9(30.7–32.7)	0.452
RDW‐CV (%), median (25°–75°)	14(13.2–15.1)	13.9(12.8–15.6)	0.488
CRP (mg/L), median (25°–75°)	1.25(0.82–6.07)	1.25(0.47–3.2)	0.258
Start Therapy			
Time starting therapy after transplant (days), median (25°–75°)	3.5(2.25–5.75)	2 (1–2)	<0.001
eGFR (ml/min/1.73m2), median (25°–75°)	16.2(6.8–24.5)	9.3 (6.2–12.9)	0.152
Serum creatinine (mg/dL), median (25°–75°)	3.3(2.5–6.4)	6.1 (4.5–6.8)	0.105
Hb (g/dL), median (25°–75°)	9.3(8.8–10.4)	9.4 (8.9–10)	0.955
HCT (%), median (25°–75°)	29.1(27–31.9)	28.3 (27–31.4)	0.970
MCV (fL), median (25°–75°)	96(95–98)	98 (94.2–102)	0.282
MCH (pg), median (25°–75°)	31.2(29.6–31.9)	31.5 (30.4–32.7)	0.527
MCHC (g/L), median (25°–75°)	32.4(31.2–33.1)	32.3 (31.1–32.5)	0.607
RDW‐CV (%), median (25°–75°)	14(13.2–15.1)	14.2(13.–15.7)	0.812
Ferritin (µg/L), median (25°–75°)	523(231.7–1041.2)	581(432.5–779)	0.429
TSAT (%), median (25°–75°)	29(13–45)	32.5(25.5–59.7)	0.258
Serum iron (µg/dL), median (25°–75°)	60(30.5–94)	68(41–91.7)	0.692
CRP (mg/L), median (25°–75°)	21.6(12.8–39.1)	43.9(12.9–54.8)	0.127
VitB12 (ng/L), median (25°–75°)	368(336.5–522.2)	449(359–734)	0.252
Folate (µg/L), median (25°–75°)	7(3.6–12.1)	13(3.6–13.5)	0.243
Cholesterol (mg/dL), median (25°–75°)	146.5(113.25–195.7)	173(140.5–195.5)	0.293
HDL (mg/dL), median (25°–75°)	38.5(26.5–53.5)	37(29.5–41.5)	0.629
LDL (mg/dL), median (25°–75°)	90.5(60.4–107.7)	111(71.6–135.5)	0.245
Triglycerides (mg/dL), median (25°–75°)	186.5(121.25–287)	182(150.5–272.5)	0.895

### 3.2 Hb Changes and Blood Transfusions Need over Time

**FIGURE 1:**
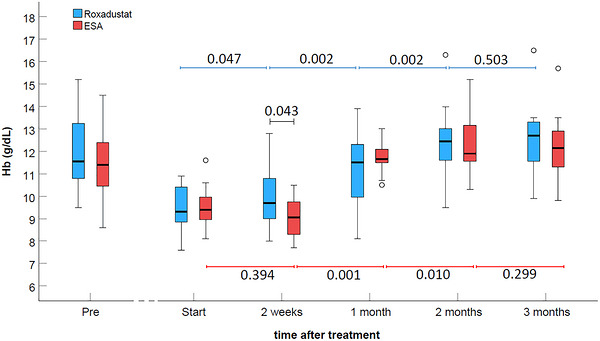
Hemoglobin changes between the two groups during the observation time. As shown in the figure, after 2 weeks, the roxadustat group (blue) showed a statistically significant increase in Hb (*p* = 0.047), whereas the ESA group (red) showed a similar effect only after 1 month (*p* < 0.001). Both groups continue to improve hemoglobin (Hb) values through the third month. A significant difference between the two groups was reported after two weeks of treatment.

Figure 1 shows the trend in hemoglobin levels over the observation period. After two weeks, the roxadustat group showed a significant improvement in Hb (*p* = 0.047) compared with baseline; the difference between the two groups was also statistically significant at this time (*p* = 0.043). In contrast to the roxadustat group, patients in the ESA group did not achieve a significant improvement in Hb; additionally, 62.25% required at least one transfusion, representing a significantly higher risk (Odds Ratio 7.222 [1.440‐36.224]) than in the roxadustat group (18.8%) (Table 2). These variations were accompanied by differences in HCT values after two weeks (*p* = 0.059), with greater increases in the roxadustat group despite fewer blood transfusions. No significant differences were observed in the other parameters (MCV, MCH, MCHC, and RDW‐CV) throughout the follow‐up. After one month, both groups showed a significant increase in Hb (*p* = 0.002 for the roxadustat group and *p* = 0.001 for the ESA group), and after two months as well (*p* = 0.002 for the roxadustat group and *p* = 0.010 for the ESA group). After three months, no significant differences were observed between the second and third months in either group (Figure 1).

**TABLE 2:** Comorbidities episodes after therapy. No significant difference was encountered between the two groups. A significant difference is noted between the two groups regarding the need for blood transfusions.

	Roxadustat	ESA	*p* value	Odds Ratio
Cardiovascular episode n° (% in group)	0(0)	2(12.5)	0.484	0.46(0.31–0.68)
Tromboembolic episode n° (% in group)	0(0)	1(6.25)	0.993	0.48(0.33–0.69)
Rejection episode n° (% in group)	2(12.5)	0(0)	0.484	0.46(0.31–0.68)
Infection episode n° (% in group)	3(20)	5(31.25)	0.685	1.81(0.35–9.45)
Blood transfusion after transplant n° (% in group)	3(18.8)	10(62.5)	0.029	7.22(1.44–36.22)

### 3.3 Kidney Function

A significant result was found only for the variation from the start to one month for serum creatinine (*p* = 0.05) and for eGFR (*p* = 0.012), (serum creatinine median 1.5 [1–2.4] mg/dL with eGFR median 28.7 [26.6–73.9] mL/min/1.73m2 for the roxadustat group at one month; serum creatinine median 2 [1.4–2.5] mg/dL with eGFR median 35.6 [25.8–45] mL/min/1.73m2 for the ESA group at one month). At the end of the three‐month observation period, no significant differences were observed between the two groups in serum creatinine or eGFR. This result is partly explicable by the presence in the roxadustat group of two patients who experienced an early rejection episode.

### 3.4 Iron Metabolism and Inflammatory Status After 1 Month

**FIGURE 2:**
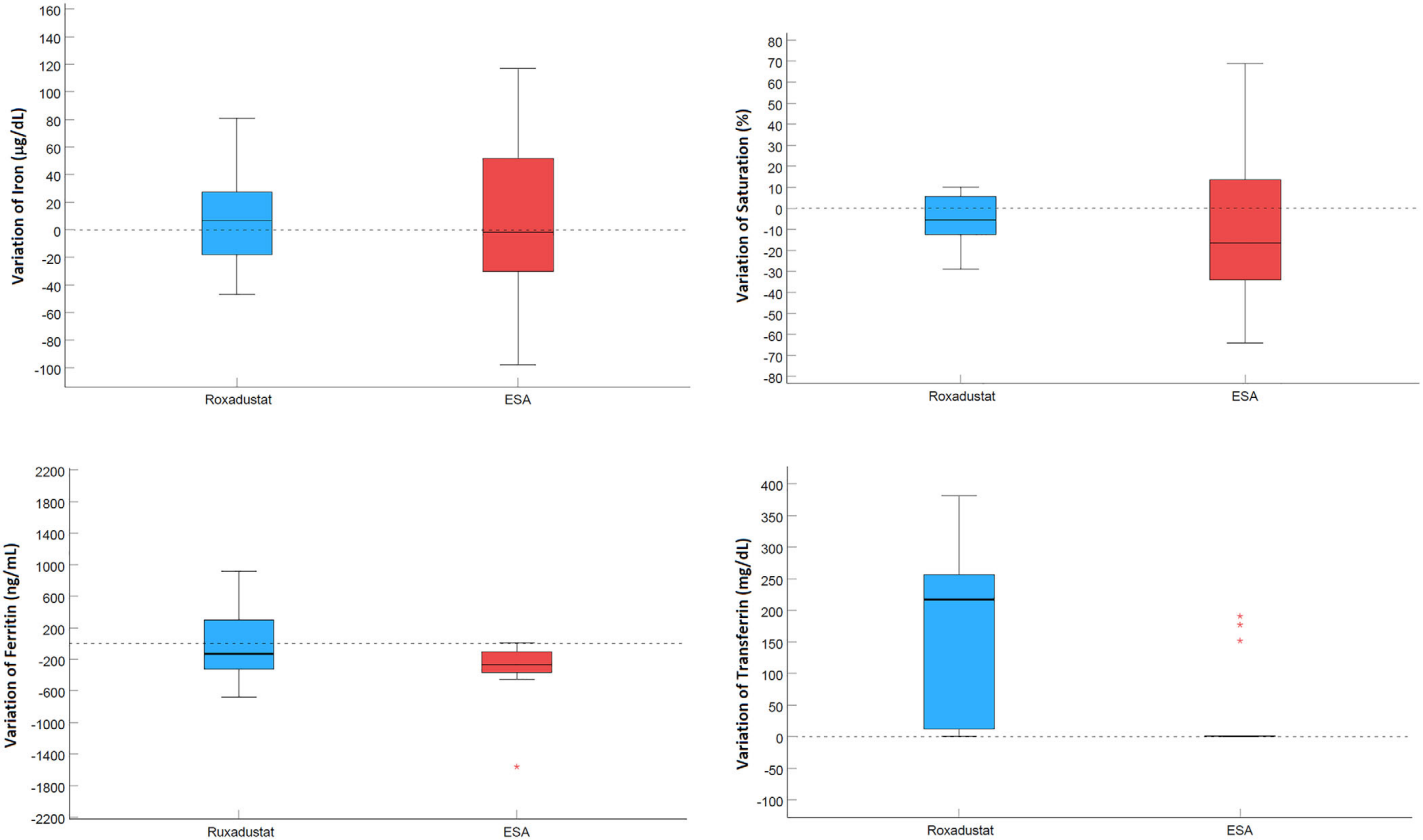
Variations in iron metabolism from the start to one month for iron, ferritin, TSAT, and transferrin. A significant difference in transferrin values (*p* = 0.007) is observed between the roxadustat (blue) and ESA (red) groups. The other variables do not reach statistical significance, although it is crucial to note a positive median change in iron values. These data demonstrate higher iron bioavailability in the roxadustat group than in the ESA group.

Regarding iron metabolism and inflammatory status, we analyzed serum iron, transferrin, transferrin saturation (TSAT), serum ferritin, and C‐reactive protein (CRP). No differences were observed between the two groups at baseline, as summarized in Table 1. After 1 month, the analysis of iron status demonstrates an increased iron availability in roxadustat group by reducing ferritin (median ‐128.5 [−323, +385]) µg/L, which in turn mobilizes stored iron (median +6.5 [−19; +29.7]), TSAT (median −5.5 [−12.7; +5.7]), leading to higher levels of transferrin (median +217.4 [+7.7; +257.6]), as reported in roxadustat use [14], Figure 2. In contrast, the same Hb variation in the ESA group after one month (no difference between two groups *p* = 0.533) determined a median reduction of iron (median ‐2 [−33; +53.7]), TSAT (median ‐16.5 [−34.5; +16.2]), ferritin (median −264.5 [−367; −95]), and a substantial no modification of transferrin (median +0.8 [+0.3; +1.3]) as in sideropenic anemia. Analysis of the percentage of variation and comparison of the two groups reveal a significant difference in transferrin values (*p* = 0.007), whereas ferritin does not reach statistical significance (*p* = 0.07). Both groups presented three patients who received a dose of 500 mg of carboxymaltose ferric.

Statistical differences were found in the CRP analysis only after two weeks (*p* = 0.015) (the roxadustat group median 1.0 [0.4–3.2] mg/L; the ESA group median 3.7 [1.5–11] mg/L). These results could be explained by the higher number of infection episodes experienced by the ESA group (5 vs. 3). Both groups, after one month, presented a reduction in terms of CRP [the roxadustat group median ‐17.5 (‐36.4; 7.1); the ESA group median ‐37.6 (−49.6; −21.9)]. These reductions did not reach statistical significance (*p* = 0.082), as confirmed by percentage analysis (*p* = 0.304).

We also analyzed vitamin B12 and folate levels during this period and found no statistically significant difference between the two groups. Only the difference for vitamin B12 resulted statistically significant (*p* = 0.047, median 573 ng/L for the roxadustat group, median 362 ng/L for the ESA group).

### 3.5 Blood Lipid Changes After 1 Month

We also analyzed the lipid status in both patient groups. There were no significant differences between the two groups at baseline, as measured by cholesterol, HDL, LDL, and triglycerides. After one month, we found a significant difference for cholesterol (roxadustat 141[106–189] mg/dL; ESA 193.5 [172.7–231.5] mg/dL [*p* = 0.007]) and LDL (roxadustat 86 [51.4–114.5] mg/dL; ESA 116 [113.5–149.5] mg/dL [*p* = 0.007]), while tryglicerides not reach the statistical significance (*p* 0.082). In absolute and percentage terms, only the change in triglyceride levels from baseline to one month was statistically significant (*p* = 0.027). At the end of the study, no statistically significant differences were observed in any parameter between the two groups. These results may depend on the administration of omega‐3 and statin at discharge.

### 3.6 Safety and Adverse Effects

One episode of TIA, two episodes of leukopenia, and one episode of gout were reported in the roxadustat group. No allergic reactions to therapy have been reported during the study. No difference was observed between the two groups in cardiovascular, thromboembolic, or rejection events; a nonsignificant trend for increased infection episodes was noted in the ESA group (Odds ratio 1.81, *p* = 0.685), which may be partially attributable to the higher proportion of patients with a history of immunological nephropathy in that group (Table 2).

## 4 Discussion

In this study, a standard dose of roxadustat (based on subject weight) and a high dose of ESA (10000 Units, three times per week) were compared in an early posttransplant population (<7 days posttransplant). According to multiple studies [15, 16], which demonstrate the non‐inferiority of roxadustat compared to ESA in CKD, and in posttransplant patients [9, 10, 11, 12, 13] the efficacy and safety of roxadustat compared to ESA were tested when the inflammatory status in KTRs is higher, during the first days after transplant. In these patients, multiple factors (surgical intervention, delayed graft function, immunosuppressive drugs, allograft rejection, and infections) can contribute to ESA resistance by increasing the inflammatory status. PTA is an important complication after KTRs management, because, as reported in various studies [17, 18, 19, 20] it is associated with higher rates of all‐cause mortality, graft failure, and congestive heart failure. Furthermore, patients with PTA who receive multiple blood transfusions increase their risk of alloimmunization [21, 22, 23], early and late graft loss [24, 25, 26], and the wait time for future re‐transplantation [23, 24]. For these reasons, the timing and efficacy of erythropoiesis stimulation after transplantation are key factors in addressing PTA. In this study, we reported a group of consecutive patients who received both ESA and roxadustat early after transplantation, considering it the most critical to address the need for blood transfusions. The starting point of therapy has been anticipated forward to a few days after transplantation, when the patient needs it most, because of the surgery and the inflammation status, unlike the other works [9, 13].

Despite delayed initiation and a different unfavorable vintage (with more transfusions before starting therapy) in the Roxadustat group, Roxadustat rapidly improved Hb levels after 2 weeks, significantly reducing the subsequent need for blood transfusions.

In Guenal et al. [13]., roxadustat treatment led to a rapid and significant increase in Hb levels, with statistically significant results as early as two weeks, and was also associated with a significant reduction in blood transfusions. Furthermore, after one month of therapy, we observed a favorable lipid profile in patients treated with roxadustat, which, in part, corroborates results from studies of roxadustat in patients with CKD [27], although Li et al. [9] didn't find the same results. The small patient sample in these studies might explain this difference.

Absolute iron deficiency and functional iron deficiency are the other two causes of PTA. The first is due to reduced iron stores, whereas the second is caused by an inflammatory process that limits iron release, even in the presence of adequate iron stores. As shown in an animal study [28] HIF regulates the iron duodenal absorption and transport to tissues. Already Kong et al. [10]. found a significant increase in iron, transferrin, and TIBC four weeks after starting roxadustat treatment. Similarly, we observed improved iron utilization in the roxadustat group compared with the ESA group, even in patients with high inflammatory status immediately posttransplantation. At month 1, after starting therapy, in the roxadustat group, the median positive change in serum iron and transferrin indicates increased iron availability, consistent with the observed increase in Hb values. In contrast, in the ESA group, no substantial variation was reported in transferrin and serum iron levels. Ferritin decreased in both groups, with no significant difference between them.

To our knowledge, this study is the roxadustat and ESA immediately following kidney transplantation in patients with inflammation. Despite this, we acknowledge the limitations of our work, primarily arising from its retrospective design, low sample size, and the absence of other markers of inflammatory status/iron metabolism (e.g., hepcidin).

However, we demonstrate a benefit of roxadustat in a difficult‐to‐treat, frail population, showing that Roxadustat is an effective and safe treatment that increases hemoglobin levels and reduces the need for blood transfusions in this setting. His administration should be considered for PTA management, particularly in patients with high inflammatory status.

## Conflicts of Interest

The authors declare no conflicts of interest.

## Funding

This work was supported by University of Turin, Department of Medical Sciences.

## Data Availability

The data that support the findings of this study are available on request from the corresponding author. The data are not publicly available due to privacy or ethical restrictions.
